# ApoE4 Astrocytes Secrete Basement Membranes Rich in Fibronectin and Poor in Laminin Compared to ApoE3 Astrocytes

**DOI:** 10.3390/ijms21124371

**Published:** 2020-06-19

**Authors:** Abby Keable, Ronan O’Neill, Matthew MacGregor Sharp, Maureen Gatherer, Ho Ming Yuen, David Annandale Johnston, Roy Oliver Weller, Roxana Octavia Carare

**Affiliations:** Faculty of Medicine, University of Southampton, Southampton SO16 6YD, UK; A.C.Keable@soton.ac.uk (A.K.); ron1e11@soton.ac.uk (R.O.); m.t.sharp@soton.ac.uk (M.M.S.); M.Gatherer@soton.ac.uk (M.G.); H.M.Yuen@soton.ac.uk (H.M.Y.); D.A.Johnston@soton.ac.uk (D.A.J.); roy.weller@ntlworld.com (R.O.W.)

**Keywords:** apolipoprotein E4 astrocytes, basement membranes, correlative light and electron microscopy, millifluidics

## Abstract

The accumulation of amyloid-β (Aβ) in the walls of capillaries and arteries as cerebral amyloid angiopathy (CAA) is part of the small vessel disease spectrum, related to a failure of elimination of Aβ from the brain. Aβ is eliminated along basement membranes in walls of cerebral capillaries and arteries (Intramural Peri-Arterial Drainage—IPAD), a pathway that fails with age and ApolipoproteinEε4 (ApoE4) genotype. IPAD is along basement membranes formed by capillary endothelial cells and surrounding astrocytes. Here, we examine (1) the composition of basement membranes synthesised by ApoE4 astrocytes; (2) structural differences between ApoE4 and ApoE3 astrocytes, and (3) how flow of Aβ affects Apo3/4 astrocytes. Using cultured astrocytes expressing ApoE3 or ApoE4, immunofluorescence, confocal, correlative light and electron microscopy (CLEM), and a millifluidic flow system, we show that ApoE4 astrocytes synthesise more fibronectin, possess smaller processes, and become rarefied when Aβ flows over them, as compared to ApoE3 astrocytes. Our results suggest that basement membranes synthesised by ApoE4 astrocytes favour the aggregation of Aβ, its reduced clearance via IPAD, thus promoting cerebral amyloid angiopathy.

## 1. Introduction

The accumulation of amyloid-β (Aβ) peptides in the walls of cerebral capillaries and arteries as cerebral amyloid angiopathy (CAA) is a key element in the spectrum of small vessel diseases [1,2]. The prevalence of the APOE polymorphic alleles in the general population is of 8.4% for ε2, 77.9% for ε3 and 13.7% for ε4 [3]. Apart from increasing age, the possession of apolipoprotein E ε4 (ApoE4) genotype is the highest risk factor for CAA [4].

Astrocytes produce and release apolipoprotein E and lipoproteins in the extracellular space [5], whereas neurons metabolise cholesterol. In the presence of ApoE4, the cerebral lipidomes and transcriptomes are altered, particularly affecting the proteasome-autophagy systems [6]. In Alzheimer’s disease (AD) brains, possession of ApoE4 is associated with thin basement membranes and breakdown of the blood-brain barrier (BBB) [7]. Furthermore, the breakdown of the BBB accompanied by cognitive deficits has also been shown in living individuals in the absence of amyloid deposits [8]. The possession of ApoE4 genotype is also associated with a lower capacity for clearance of interstitial fluid and soluble proteins from the brain [9]. Taken together, a variety of experimental studies using rodents and observations in humans demonstrate that ApoE4 promotes the aggregation of Aβ and impairs its clearance, while accelerating the development of dementia, in particular after cerebrovascular events [10,11].

We have demonstrated that cerebrovascular basement membranes are the pathway of drainage of interstitial fluid and soluble amyloid beta (Aβ) as intramural periarterial drainage (IPAD) [12,13]. Transgenic mice that overexpress ApoE4 have a disrupted pattern of Aβ clearance via IPAD [14]. The age-related changes in the extracellular matrix and cerebrovascular basement membranes lead to a slowing of IPAD and they are key in the pathogenesis of CAA [15]. So far there has been little evidence for the changes in extracellular matrix in the presence of ApoE4 when compared to ApoE3.

The objective of the present study is to test the hypothesis that ApoE4 astrocytes differ from ApoE3 in ways that ApoE4 astrocytes would promote the development of CAA and Alzheimer’s disease. We test this hypothesis by (1) comparing the nature of the extracellular matrix (ECM) (basement membrane) formed by ApoE4 and ApoE3 astrocytes in cell culture, by (2) examining the structural differences between ApoE4 and ApoE3 astrocytes, and (3) determining how the flow of Aβ over the cells differentially affects ApoE4 and ApoE3 astrocytes.

We show that ApoE4 astrocytes synthesise more fibronectin, possess smaller processes, and become rarefied when Aβ flows over them when compared to ApoE3 astrocytes. We discuss how our results suggest that basement membranes synthesised by ApoE4 astrocytes favour the aggregation of Aβ and impair its clearance via IPAD pathways to promote CAA and Alzheimer’s disease.

## 2. Results

### 2.1. ApoE4 Astrocytes Have a Reduced Rate of Proliferation Compared to ApoE3 Astrocytes and Synthesise Different Basement Membranes

**Figure 1:**
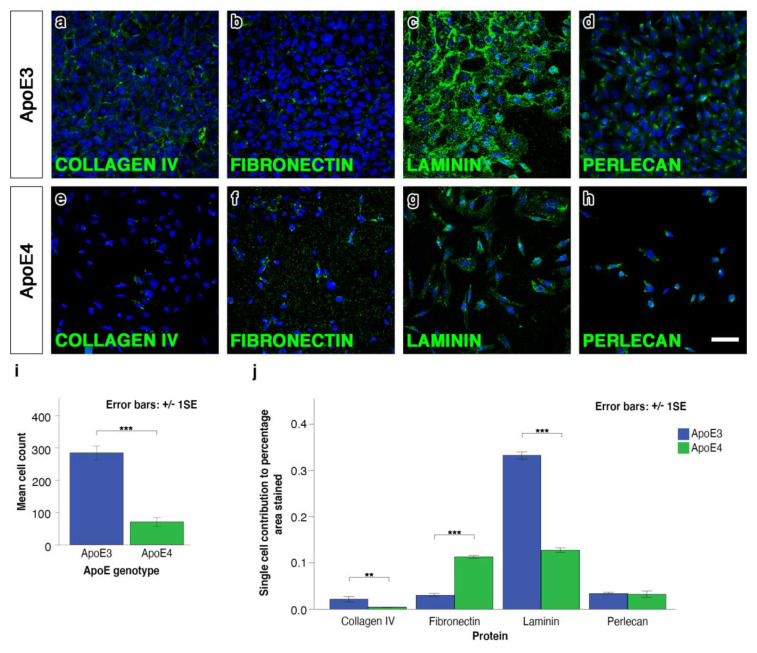
Total cell count, immunostaining and quantification for basement membrane proteins after 72 h in culture. Representative threshold images used for calculating the percentage area staining for ApoE3 and ApoE4 astrocytes are shown in (**a**) and (**e**) for collagen IV, in (**b**) and (**f**) for fibronectin, in (**c**) and (**g**) for laminin, in (**d**) and (**h**) for perlecan where the immunostained proteins are shown in green and 4′,6-diamidino-2-phenylindole (DAPI) shown in blue, scale bar 50 μm. (**i**) The mean cell count for ApoE4 astrocytes is significantly lower when compared to ApoE3 astrocytes (*** *p* < 0.001). (**j**) The results were based on the average number of DAPI stained nuclei in three non-overlapping images over six independent experimental runs. When the percentage area stained was divided by the cell count to calculate the individual cell contribution to the total percentage area staining for basement membrane proteins, there was significantly more fibronectin (*** *p* < 0.001), less laminin (*** *p* < 0.001), and less collagen IV (** *p* < 0.01) in the ApoE4 cells when compared to ApoE3 cells. Average percentage area stained was calculated from three fields of view per coverslip and a total of three coverslips per protein over three independent experiments. Error bars represent the standard error of the mean and significance is indicated, as follows ** *p* < 0.01, *** *p* < 0.001.

The mean cell count in cultured astrocytes at 72 h was calculated from eight-bit black and white images of the DAPI stained nuclei of ApoE3 and ApoE4 astrocytes *p* < 0.001 and showed significantly reduced number of ApoE4 astrocytes when compared to ApoE3 cells (Figure 1i). There were almost three times more ApoE3 astrocytes after culturing for 72 h as compared to ApoE4 astrocytes. No extracellular matrix proteins were observed in either genotype before 72 h. At 72 h after culture, there were differences in the composition of extracellular matrix between the astrocytes. By measuring mean percentage area staining per cell, we found ApoE4 cells secreted significantly less collagen IV and laminin than ApoE3 cells (*p* < 0.001) (Figure 1a,c,e,g,j). ApoE4 astrocytes secreted significantly more fibronectin and there was no difference in the secretion of perlecan (Figure 1d,h,j).

### 2.2. ApoE4 Astrocytes Have Altered Cellular Morphology With Fewer Cellular Processes

**Figure 2:**
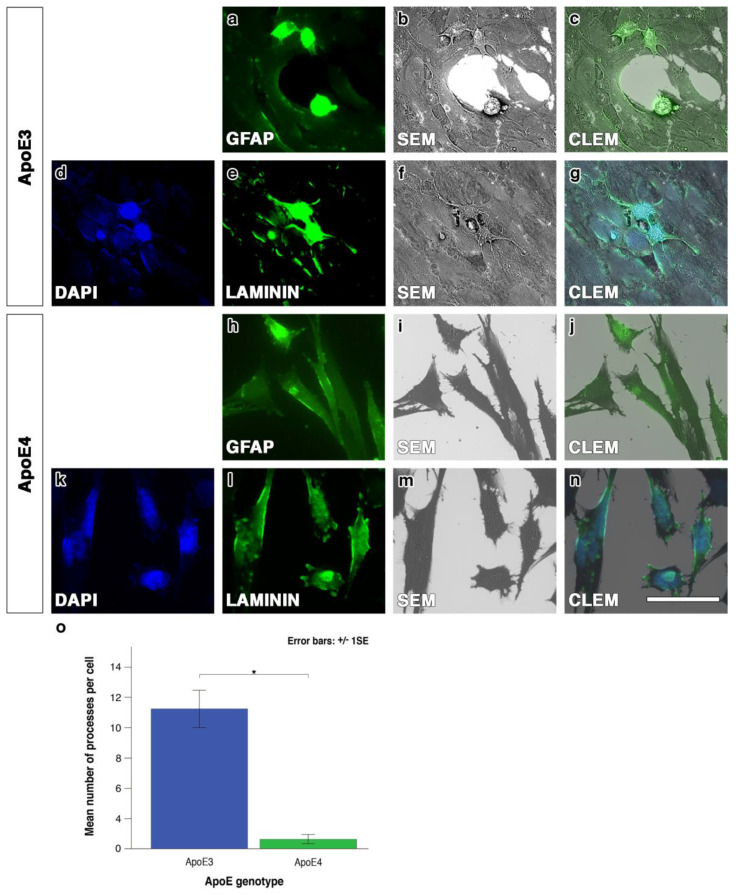
The appearance and quantification for ApoE3 and ApoE4 astrocytes with Correlative Light and Electron Microscopy (CLEM). Immunostaining with GFAP provided evidence the cells were astrocytes (**a**,**h**). Laminin immunostaining with DAPI nuclear stain (**d**) for ApoE3 astrocytes showed strong laminin immunolabelling of the cell bodies and processes (**e**) and the SEM (**b**,**f**) and CLEM features (**c**,**g**) showed a round/hexagonal/pentagonal shape of the cell body. ApoE4 astrocytes immunolabelled with laminin (**l**) and DAPI nuclear counterstain (**k**) showed very little immunolabelling of cell processes. The SEM (**i**,**m**) and CLEM (**j**, **n**) demonstrated the elongated shape of the cell bodies for ApoE4 astrocytes. (**o**) There was a significant reduction in the number of processes in ApoE4 astrocytes as compared to ApoE3 (*p < 0.001). Green: immunostaining with GFAP or laminin. Blue: DAPI counterstain. Results were based on data gathered over three independent experiments and error bars represent the standard error of the mean, scale bar: 40 µm.

With its capability of detecting structures of 15 nm, the correlative light and electron microscopy (CLEM) demonstrated the detailed morphology of both ApoE3 and ApoE4 astrocytes. We first confirmed that the cultured cells were indeed astrocytes, as they expressed Glial Fibrillary Acidic Protein (GFAP), (Figure 2a,h) and had the morphological appearance of astrocytes, as observed by scanning (Figure 2b,i) and correlative light electron microscopy (Figure 2c,j). We then assessed the morphology of the cells by comparing their overall appearance and counting the number of cellular processes using CLEM (Figure 2c,g,j,n). The mean number of cellular processes per cell was significantly lower in ApoE4 astrocytes when compared to ApoE3 astrocytes (*** *p* < 0.001) (Figure 2o). Immunostaining with laminin and DAPI revealed ApoE3 astrocytes with fuller cell bodies pentagonal/hexagonal in shape with longer, thicker cellular processes (Figure 2d,e,f,g). Conversely, ApoE4 astrocyte cell bodies appeared to be elongated with short cellular processes (Figure 2k,l,m,n).

### 2.3. ApoE4 Astrocytes Are More Sensitive to Flow of Aβ 

We first tested whether flow alone had an effect upon the ApoE4 astrocytes when compared to static conditions. After calibrating the peristaltic pump, pilot experiments were conducted without the addition of amyloid beta to investigate how well the cells grow within the Quasi Vivo system and ascertain if flow alone influences the cells. The astrocytes grew well inside the Quasi Vivo system, indicating that the polymer is biocompatible and does not adversely affect the cells. The astrocytes expressed GFAP, regardless of the culture conditions. We chose to test the effects of flow alone on ApoE4 astrocytes, as they appeared less robust in their pattern of growth. A qualitative comparison of cell densities between the static and flow cultures of ApoE4 astrocytes showed very little difference in our pilot experiments.

**Figure 3:**
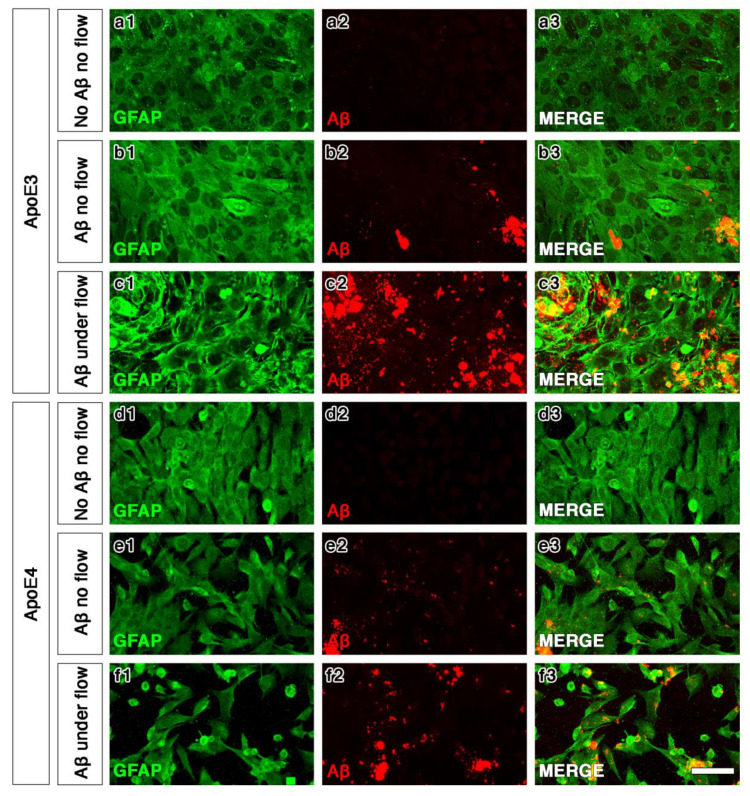
Confocal images of ApoE3/ApoE4 astrocytes after incubation with no Aβ (**a1**–**a3**,**d1**–**d3**), Aβ for 24 h in static conditions (**b1**–**b3**,**e1**–**e3**) or Aβ for 24 h with flow (**c1**–**c3**,**f1**–**f3**) in the Quasi Vivo system. Both genotypes showed a similar distribution and aggregation of Aβ under no flow (**b2**,**e2**) that became more widespread under flow (**c2**,**f2**). Flow conditions did not affect the appearance of ApoE3 astrocytes (**b1**,**c1**), but did affect ApoE4 astrocytes with a more sparse arrangement under flow (**e1**,**f1**). Colour representations are: green: GFAP (**a1**,**b1**,**c1**,**d1**,**e1**,**f1**); red: Aβ (**a2**,**b2**,**c2**,**d2**,**e2**,**f2**); and, overlay (**a3**,**b3**,**c3**,**d3**,**e3**,**f3**). Scale bar: 75 µm.

In static cultures, Aβ was observed after its application on both ApoE3 and ApoE4 astrocyte cell cultures. The Quasi Vivo millifluidic system enhanced the pattern of distribution of Aβ in both ApoE3 and ApoE4 cells (Figure 3c3,3f3) when compared to no flow (Figure 3b3,3e3). The flow of Aβ over ApoE4 astrocytes resulted in a disturbance in their pattern, with their rarefaction (Figure 3f1–3) as compared to the static application of Aβ (Figure 3e1–3).

## 3. Discussion

In our study, every ApoE4 astrocyte expressed more than twice the amount of fibronectin and less than half of the amount of laminin compared to ApoE3 astrocytes. We used immunocytochemistry to analyse the levels of protein rather than transcriptomics or any other gene analysis, because there is inconsistency between the expression of mRNA expression and proteins among the isoforms of ApoE [16]. Furthermore, from the perspective of pathogenesis of CAA, it is the extracellular composition of the basement membranes that is relevant rather than a total amount of protein that could include an intracellular compartment.

Our results suggest that ApoE4 astrocytes synthesise a basement membrane that promotes the aggregation of Aβ, similar to how TGFβ promotes the synthesis of fibronectin rich basement membranes and cerebrovascular amyloid [17,18]. It is possible that a basement membrane that is rich in fibronectin would sequester and favour the aggregation of Aβ [19].

Astrocytic ApoE4 impairs the clearance of Aβ in vivo [20] and there is reduced lysosome degradation of Aβ within the ApoE4 astrocytes [20]. Inducible models of ApoE3/4 demonstrate that ApoE4 is associated with enhanced seeding of Aβ and its reduced clearance from the parenchyma [10]. The fibronectin rich glial basement membranes would explain why specifically IPAD of Aβ is impaired in the presence of ApoE4 [14] and the higher prevalence of CAA in capillary walls (CAA Type 1) for ApoE4 positive individuals [21,22]. Pial cells influence the amount and type of laminin synthesized by astrocytes [23,24]. Although we assessed single cell astrocyte cultures in our study and the immunocytochemistry was performed for pan-laminin, the reduction in the laminin secreted by ApoE4 astrocytes suggests there may be consequences upon the nature of the pial-glial basement membranes [24]. Because the pial-glial basement membranes are the conduit for convective influx/glymphatic entry of cerebrospinal fluid into the brain, their altered composition in ApoE4 individuals may be associated with a disruption in the equilibrium of the exchange between the cerebrospinal and interstitial fluids [25,26].

Correlative light and electron microscopy (CLEM) refers to a technique that combines an optical microscope, typically fluorescence, with an electron microscope. The integrated CLEM microscopy used in this study (Delphi) is made up of a table top scanning electron microscope combined with an inverted fluorescence microscope, ideal for the examination of the morphology of astrocytes and their processes, as it is able to resolve structures of 15 nm [27]. Using CLEM, we demonstrate here that ApoE4 astrocytes have shorter and fewer processes when compared to ApoE3 astrocytes. Our assessment of an astrocytic process was made based on the connection with the soma and a length of a minimum of 100 nm, as most astrocytic processes are over 4µm [28,29]. This suggests that ApoE4 astrocytes may be less able to perform their function, in particular that of maintaining the integrity of the blood brain barrier (BBB) [30]. It has already been shown that, in adult ApoE4 transgenic mice, there is an activation of the cyclophlin-A-NFκB-metalloprotease-9 pathway, leading to a disruption of BBB integrity and it is possible that the astrocytes of the ApoE4 mice are not physiologically capable of maintaining the integrity of the BBB [31]. Furthermore, it was recently shown that the number and area of CD31-positive microvessels in the neocortex and corpus callosum of ApoE4 mice is reduced compared to ApoE3 and age matched wild type controls [32]. The disruption in the BBB has also been demonstrated in ApoE4 individuals who also show early cognitive deficits in the absence of amyloid deposits [8]. Astrocytic laminin is essential for the development of the BBB and we show here that ApoE4 astrocytes secrete a lower amount of laminin when compared to ApoE3 astrocytes. Collagen IV is necessary for vessel wall integrity and the low synthesis of collagen IV by ApoE4 astrocytes observed in our study suggests that ApoE4 individuals may have fragile vessels, possibly more capillary CAA or small vessel disease [33,34]. P301S tau transgenic mice bred on a human ApoE4 background have significantly higher tau levels in the brain and a greater extent of somatodendritic tau redistribution by three months of age as compared with P301S tau mice bred on a human ApoE3 background or ApoE null background mice [35]. It might be that neurons in ApoE4 positive brains require a higher number of synapses to compensate for the fewer astrocytic-neuronal connections [20].

The Quasi Vivo millifluidics system is perfectly suitable for the study of IPAD, as it allows flow via a peristaltic pump over cell cultures [36]. We calibrated the pump and adapted the system to flow Aβ at a concentration of 100 nm, which is several orders of magnitude lower and closer to the physiological range as compared to most in vitro studies. We show that Aβ accumulates more in both ApoE3 and ApoE4 during flow when compared to its application on static cultures. This might be due to the physical effects of movement. The ApoE4 astrocytes are rarefied after flow as compared to static cultures, which suggests that Aβ itself has a detrimental effect specifically on ApoE4 astrocytes.

In conclusion, our study using novel correlative light and electron microscopy as well as a millifluidic system, in addition to conventional immunocytochemistry, demonstrates that ApoE4 astrocytes synthesise more fibronectin and have shorter processes when compared to ApoE3 astrocytes. The flow of Aβ resulted in detrimental effects upon the ApoE4 cells in culture, although we have not performed any cell death assays. One limitation of the study is that the cells used in this paper (immortalized cells from knock-in mice produced in 2005) were not checked for their levels of expression of the transgenes, although we did use them for the production of ApoE3/4 lipoproteins for a different study. The results of the present study support the working hypothesis that ApoE4 astrocytes secrete a basement membrane that favours the aggregation of Aβ within its intramural periarterial drainage pathway and adds to the present body of evidence for a mechanistic explanation of breakdown of the blood brain barrier in the presence of ApoE4.

## 4. Materials and Methods

### 4.1. Ethical Approval

The project received ethical approval ERGO 21045 on 28 June 2016 from University of Southampton Ethics and Research Governance Online (ERGO).

### 4.2. Culturing of Human ApoE3 and ApoE4 Astrocytes

Immortalized human ApoE3 knockin and ApoE4 knockin cell lines generated from primary astrocyte cultures derived from ApoE knock-out mice expressing different homozygous human ApoE isoforms under the control of endogenous mouse ApoE promoter [37,38], were generously produced and donated by Prof David Holtzman (Washington University School of Medicine in St. Louis, MO, USA). The cells were cultured in advanced DMEM medium supplemented with 1 mM sodium pyruvate, 10% fetal bovine serum and 100 mg gentamycin and maintained at 37 °C in a 5% CO_2_ incubator. All cell culture products were purchased from Thermo Fisher Scientific. Unless stated otherwise, the following experimental procedures were performed at room temperature with each wash step repeated three times.

### 4.3. Processing Cultured Astrocytes for Confocal Microscopy

Astrocyte cultures were plated on 12 mm round coverslips in a 24-well plate at a density of 0.5 × 10^5^ cells/well and fixed in 4% paraformaldehyde (PFA) in 0.01 M phosphate buffered saline (PBS) 72 h after initial plating. The cells were washed in PBS, blocked with 15% normal goat serum (NGS) (G9023, Sigma) for one hour and then incubated overnight with primary antibodies at 4 °C (Table 1). After a further wash in PBS, cells were incubated with Alexa Fluor 488 conjugated goat anti-rabbit secondary antibody (A11034, Invitrogen, Carlsbad, CA, USA) for one hour, washed again in PBS and then incubated with 2 µg/mL 4′,6-diamidino-2-phenylindole (DAPI) (Thermo Fisher, D1306, Paisley, UK) for 10 min. The coverslips were rinsed with PBS (×1), mounted onto slides with Mowiol and examined using a confocal microscope (SP8, Leica Microsystems, UK). These experiments were repeated three times and n refers to the number of coverslips imaged.

### 4.4. Processing Cultured Astrocytes for Correlative Light and Electron Microscopy (CLEM)

The DELPHI microscope is the result of the merging of two Dutch companies: Delmic, integrated microscopy solutions and Phenom World that produce benchtop electron microscopes [27]. The Delphi system integrates fluorescence with a scanning electron microscope, as the epi-fluorescence microscope is placed directly under the electron column.

Astrocyte cultures were plated onto Indium tin oxide (ITO) coated coverslips in a 12-well plate at a density of 0.5 × 10^5^ cells/well and fixed with 2.5% paraformaldehyde and 0.25% glutaraldehyde in 0.01M PBS 72 h after initial plating. The cells were rinsed with PBS (×1), blocked with 15% NGS for 30 min then incubated overnight at 4 °C with either anti-laminin (L9393, Sigma) or anti-GFAP (GFAP) (Z0334, Dako) primary antibody (Table 1). After washing in PBS, the cells were incubated with Alexa Fluor 488 conjugated goat anti-rabbit secondary antibody (A11034, Invitrogen) for one hour. The primary and secondary antibodies were both diluted with 15% NGS to ensure specificity by continuous blocking. After incubation, the cells were quickly rinsed with distilled water, exposed to cupric sulphate (10 mM copper sulphate in 50 mM ammonium acetate, pH 5.0) for 10 min to quench autofluorescence, quickly rinsed again with distilled water, and then stained with 2 µg/mL DAPI for 10 min. After washing with PBS and quickly rinsing in 70% ethanol, cells were dehydrated via ethanol dilution series: 70%, 80% and 90% (5 min), 100% (2 × 15 min). Coverslips were air dried overnight in the dark and then mounted onto carrier rings with carbon tape and examined by CLEM (Delphi microscope, Phenom world, Delft, The Netherlands). These experiments were repeated three times (*n* = 3). 

### 4.5. Testing the Effect of Amyloid Beta on Cultured Astrocytes

#### 4.5.1. Preparation of Amyloid Beta

In a healthy brain, the Aβ concentration is thought to be in the range of 2–3 nM [39], although this is significantly higher in an AD brain [40]. To replicate the burden of amyloid in AD brain, 100 nM Hilyte 555 Aβ1–40 (Anaspec, AS-60492-01) was added to the culture media at the start of the 24 h flow experiments. The Hilyte 555 Aβ1–40 was supplied as a lyophilised powder and reconstituted with 1% ammonium hydroxide to a final concentration of 200 μM. A concentration of 100 nM was achieved by adding 1 μL of the 200 μM stock of amyloid beta per 1 mL culture media. 100 nM was as close to physiologically relevant as possible whilst still being detectable by confocal microscopy.

#### 4.5.2. Static Amyloid Beta Experiments

ApoE3 or ApoE4 astrocytes were plated onto poly-l-lysine coated coverslips in a 24-well plate at a density of 1 × 10^5^ cells per well. The cells were allowed to grow undisturbed for 24 h before the media was changed. Either normal astrocyte medium or astrocyte medium supplemented with 100 nM Hilyte 555-labelled Aβ1–40 (Anaspec) was added to the cells and left for a further 24 h. Cells were then fixed with 4% PFA for 10 min before immunostaining.

#### 4.5.3. Flowing Amyloid Beta Experiments

**Figure 4:**
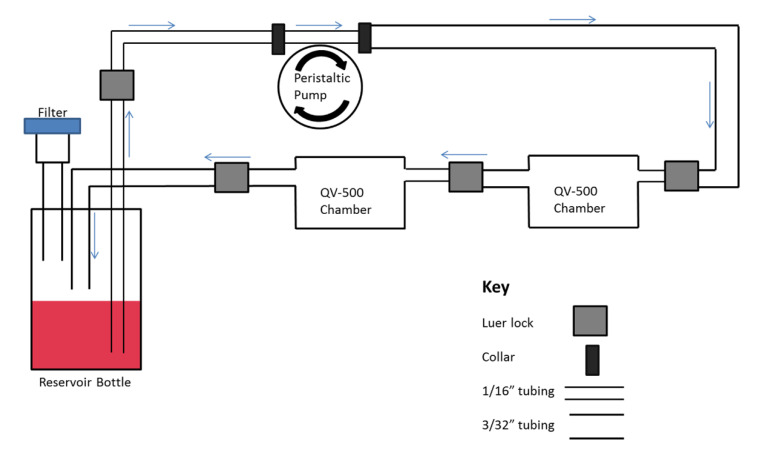
Schematic representation of a two chamber QV500 flow system. When assembled the system forms a closed recirculating loop allowing unidirectional flow at a speed determined by the peristaltic pump. The internal dimensions of the QV500 chamber are the same as a 24-well plate. The tubing and chambers are gas permeable and the reservoir bottle has an air filter attached to allow for gas exchange.

A novel interconnected cell culture flow system (Quasi Vivo, Kirkstall Ltd., Rotherham) was used to apply flow to the astrocyte cultures. The system was set up, as shown in Figure 4 A polyflex dual-head peristaltic pump (PARKER PF22X0103, Kirkstall, Sheffield, UK) was calibrated for a two-chamber set up according to the manufacturer’s instructions. ApoE3 or ApoE4 astrocytes were counted and plated on coverslips in a 24-well plate at a density of 1 × 10^5^ cells per 12 mm coverslip and left undisturbed for 24 h to allow cells to settle and attach. After 24 h, two coverslips with ApoE3 astrocytes were transferred into the QV500 chamber for a flow experiment (with either normal astrocyte medium or astrocyte medium supplemented with 100 nM Hilyte 555-labelled Aβ1–40) and left to run for 24 h. The experiment was then repeated with ApoE4 astrocytes Each ApoE genotype was loaded into the flow system and run independent of the other. The coverslips were then removed from the system and the cells were fixed with 4% PFA before immunostaining. The flow rate was based upon known values for the production of CSF (0.3–0.4 mL/min) and estimated values of the production of ISF (100× less than the CSF produced by choroid plexus) in adult humans [41]. The Quasi Vivo system required a minimum of 10 mL of media to ensure continuous fluid flow with no air pockets. To avoid over confluence, all flow experiments were performed over a 24-h period. Therefore, we cycled 10 mL of media at a flow rate of 10 µL/min, equivalent to a flow speed of 1.56 × 10^−7^ m/sec at the cell level and a shear stress of 1.28 × 10^-6^ Pa or 1.28 × 10^−5^ dyn/cm^2^ [42]. Preliminary data revealed that this flow rate was non-toxic and did not adversely affect the growth or viability of the astrocytes.

#### 4.5.4. Immunostaining of Cultured Astrocytes Exposed to Amyloid Beta

After 24 h with/without flow, the coverslips were removed and fixed with 4% PFA in 0.01 M PBS for 10 min, washed with PBS and blocked with 15% NGS diluted in 0.1% triton in 0.01 M PBS for one h. The coverslips were then incubated overnight with rabbit anti-GFAP primary antibody (DAKO) at 4 °C, washed with PBS, and then incubated with goat anti-rabbit Alexa Fluor 488 secondary antibody (Invitrogen, A-21071) for one h. After washing with PBS, 2 µg/mL DAPI (Thermo Fisher, D1306) was applied for 10 min in the dark. The coverslips were then removed from the 24-well plate with curved tweezers and then mounted onto slides with Mowiol.

### 4.6. Image Analysis

#### 4.6.1. Confocal Image Analysis

Three non-overlapping z stacks were captured (area of each image 0.15 mm^2^) from the centre of each coverslip where disturbance to the cell layer was minimal. Confocal laser power and detection parameters were consistent across the ApoE genotypes. The percentage area stained for each image was calculated by threshold analysis using Image J software [43] and an average of the three values was taken. The number of cells in an image was calculated by counting the number of DAPI stained nuclei in the same field of view (387.92 µm × 387.92 µm). To adjust for the difference in cell density, the percentage area staining was divided by the number of cells to produce a value for percentage area stained per cell.

#### 4.6.2. CLEM Image Analysis

Delphi CLEM images were analysed using Image J software (NIH, USA). A manual cell counter was used to quantify the number of DAPI positive nuclei within a 350 × 400 µm area (0.14 mm^2^). The cell counter was also used to determine the number of processes found on each astrocyte seen in focus on the electron microscope. The criteria for a process to be counted in this instance were (1) it had to be directly attached to the astrocytic cell body and (2) a length of minimum 100 nm at its main branch. The number of processes counted was divided by the number of astrocytes in focus and an average was taken.

### 4.7. Statistical Analysis

Statistical analysis for comparing the number of cells per genotype was performed while using an independent samples T-test, as the data distribution was normal. In total, 21 coverslips were imaged over three experimental repeats. The ratio of basement membrane protein produced as compared to the cell density was calculated by dividing the percentage area stained by the number of cells. The data were analysed by independent T-test (*n* = 3). The distribution of the data from the analysis using CLEM was skewed, so the equivalent non-parametric Mann Whitney U test was used to compare the number of processes (*n* = 3). IBM SPSS statistics 24 was used for all analyses and the results were determined to be statistically significant if *p* < 0.05.

## 5. Conclusions

Using cell cultures, immunocytochemistry, confocal, and electron microscopy, we show that ApoE4 astrocytes have fewer processes and secrete less laminin and collagen IV, but more fibronectin when compared to ApoE3 astrocytes. The flow of Aβ using Quasi Vivo 500 millifluidic system resulted in no effect on the ApoE3 astrocytes, but it decreased the density of ApoE4 astrocytes. Our results suggest that ApoE4 astrocytes are more sensitive to Aβ and secrete a basement membrane poor in laminin and collagen IV, which likely results in the slow clearance of drainage of Aβ along the intramural periarterial drainage (IPAD) pathways, thus promoting cerebral amyloid angiopathy.

## Figures and Tables

**Table 1:** Details of primary antibodies used in the study.

Antibody Type	Antigen	Name Provided by Supplier	Supplier Details	Optimal Dilution
Primary	Collagen IV	Rabbit anti-collagen IV (ab6586)	Anti-collagen IV antibody polyclonal produced in rabbit, AbCam, Cambridge, UK	1:400
Primary	Laminin	Anti-laminin antibody produced in rabbit (L9393)	Anti-laminin antibody polyclonal produced in rabbit, Sigma Aldrich, St. Louis, MO, USA	1:200
Primary	Fibronectin	Anti-fibronectin antibody produced in rabbit (F3648)	Anti-fibronectin antibody polyclonal produced in rabbit, Sigma Aldrich, St. Louis, MO, USA	1:400
Primary	Perlecan	Perlecan antibody (H-300) sc-25848	Perlecan (H-300) rabbit polyclonal antibody 200 µg/ml, Santa Cruz Biotechnology, Heidelberg, Germany	1:400
Primary	GFAP	Anti-GFAP antibody (Z0334)	Polyclonal rabbit anti-GFAP antibody, Dako, Glostrup, Denmark	1:250
Secondary	Rabbit IgG	Alexa Fluor 488 Goat anti-rabbit (A11034)	Alexa Fluor 488 goat anti-rabbit IgG (H+L) Invitrogen, Carlsbad, CA, USA	1:200

## References

[1] Schreiber S., Wilisch-Neumann A., Schreiber F., Assmann A., Scheumann V., Perosa V., Jandke S., Mawrin C., Carare R.O., Werring D.J. (2019). Invited Review: The spectrum of age-related small vessel diseases: Potential overlap and interactions of amyloid and nonamyloid vasculopathies. Neuropathol. Appl. Neurobiol..

[2] Sweeney M.D., Montagne A., Sagare A.P., Nation D.A., Schneider L.S., Chui H.C., Harrington M.G., Pa J., Law M., Wang D.J.J. (2019). Vascular dysfunction-The disregarded partner of Alzheimer’s disease. Alzheimer’s Dement..

[3] Liu C.C., Kanekiyo T., Xu H., Bu G. (2013). Apolipoprotein E and Alzheimer disease: Risk, mechanisms and therapy. Nat. Rev. Neurol..

[4] Fryer J.D., Simmons K., Parsadanian M., Bales K.R., Paul S.M., Sullivan P.M., Holtzman D.M. (2005). Human apolipoprotein E4 alters the amyloid-beta 40:42 ratio and promotes the formation of cerebral amyloid angiopathy in an amyloid precursor protein transgenic model. J. Neurosci..

[5] Ladu M.J., Reardon C., Van Eldik L., Fagan A.M., Bu G., Holtzman D., Getz G.S. (2000). Lipoproteins in the central nervous system. Ann. N. Y. Acad. Sci..

[6] Lefterov I., Wolfe C.M., Fitz N.F., Nam K.N., Letronne F., Biedrzycki R.J., Kofler J., Han X., Wang J., Schug J. (2019). APOE2 orchestrated differences in transcriptomic and lipidomic profiles of postmortem AD brain. Alzheimer’s Res..

[7] Salloway S., Gur T., Berzin T., Zipser B., Correia S., Hovanesian V., Fallon J., Kuo-Leblanc V., Glass D., Hulette C. (2002). Effect of APOE genotype on microvascular basement membrane in Alzheimer’s disease. J. Neurol. Sci..

[8] Montagne A., Nation D.A., Sagare A.P., Barisano G., Sweeney M.D., Chakhoyan A., Pachicano M., Joe E., Nelson A.R., D’Orazio L.M. (2020). APOE4 leads to blood-brain barrier dysfunction predicting cognitive decline. Nature.

[9] Martel C.L., Mackic J.B., Matsubara E., Governale S., Miguel C., Miao W., McComb J.G., Frangione B., Ghiso J., Zlokovic B.V. (1997). Isoform-specific effects of apolipoproteins E2, E3, and E4 on cerebral capillary sequestration and blood-brain barrier transport of circulating Alzheimer’s amyloid beta. J. Neurochem..

[10] Liu C.C., Zhao N., Fu Y., Wang N., Linares C., Tsai C.W., Bu G. (2017). ApoE4 Accelerates Early Seeding of Amyloid Pathology. Neuron.

[11] Montagne A., Nation D.A., Zlokovic B.V. (2020). APOE4 Accelerates Development of Dementia After Stroke: Is There a Role for Cerebrovascular Dysfunction?. Stroke.

[12] Carare R.O., Bernardes-Silva M., Newman T.A., Page A.M., Nicoll J.A., Perry V.H., Weller R.O. (2008). Solutes, but not cells, drain from the brain parenchyma along basement membranes of capillaries and arteries: Significance for cerebral amyloid angiopathy and neuroimmunology. Neuropathol. Appl. Neurobiol..

[13] Morris A.W., Sharp M.M., Albargothy N.J., Fernandes R., Hawkes C.A., Verma A., Weller R.O., Carare R.O. (2016). Vascular basement membranes as pathways for the passage of fluid into and out of the brain. Acta Neuropathol..

[14] Hawkes C.A., Sullivan P.M., Hands S., Weller R.O., Nicoll J.A., Carare R.O. (2012). Disruption of arterial perivascular drainage of amyloid-beta from the brains of mice expressing the human APOE epsilon4 allele. PLoS ONE.

[15] Hawkes C.A., Hartig W., Kacza J., Schliebs R., Weller R.O., Nicoll J.A., Carare R.O. (2011). Perivascular drainage of solutes is impaired in the ageing mouse brain and in the presence of cerebral amyloid angiopathy. Acta Neuropathol..

[16] Conejero-Goldberg C., Gomar J.J., Bobes-Bascaran T., Hyde T.M., Kleinman J.E., Herman M.M., Chen S., Davies P., Goldberg T.E. (2014). APOE2 enhances neuroprotection against Alzheimer’s disease through multiple molecular mechanisms. Mol. Psychiatry.

[17] Wyss-Coray T., Lin C., Sanan D.A., Mucke L., Masliah E. (2000). Chronic overproduction of transforming growth factor-beta1 by astrocytes promotes Alzheimer’s disease-like microvascular degeneration in transgenic mice. Am. J. Pathol..

[18] Wyss-Coray T., Lin C., von Euw D., Masliah E., Mucke L., Lacombe P. (2000). Alzheimer’s disease-like cerebrovascular pathology in transforming growth factor-beta 1 transgenic mice and functional metabolic correlates 1. Ann. N. Y. Acad. Sci..

[19] Howe M.D., Atadja L.A., Furr J.W., Maniskas M.E., Zhu L., McCullough L.D., Urayama A. (2018). Fibronectin induces the perivascular deposition of cerebrospinal fluid-derived amyloid-beta in aging and after stroke. Neurobiol. Aging.

[20] Lin Y.T., Seo J., Gao F., Feldman H.M., Wen H.L., Penney J., Cam H.P., Gjoneska E., Raja W.K., Cheng J. (2018). APOE4 Causes Widespread Molecular and Cellular Alterations Associated with Alzheimer’s Disease Phenotypes in Human iPSC-Derived Brain Cell Types. Neuron.

[21] Shinohara M., Murray M.E., Frank R.D., Shinohara M., DeTure M., Yamazaki Y., Tachibana M., Atagi Y., Davis M.D., Liu C.C. (2016). Impact of sex and APOE4 on cerebral amyloid angiopathy in Alzheimer’s disease. Acta Neuropathol..

[22] Rannikmae K., Kalaria R.N., Greenberg S.M., Chui H.C., Schmitt F.A., Samarasekera N., Al-Shahi Salman R., Sudlow C.L. (2014). APOE associations with severe CAA-associated vasculopathic changes: Collaborative meta-analysis. J. Neurol. Neurosurg. Psychiatry.

[23] Di Russo J., Hannocks M.J., Luik A.L., Song J., Zhang X., Yousif L., Aspite G., Hallmann R., Sorokin L. (2017). Vascular laminins in physiology and pathology. Matrix Biol..

[24] Hannocks M.J., Pizzo M.E., Huppert J., Despande T., Abbott N.J., Thorne R.G., Sorokin L. (2017). Molecular characterization of perivascular drainage pathways in the murine brain. J. Cereb. Blood Flow Metab..

[25] Albargothy N.J., Johnston D.A., MacGregor-Sharp M., Weller R.O., Verma A., Hawkes C.A., Carare R.O. (2018). Convective influx/glymphatic system: Tracers injected into the CSF enter and leave the brain along separate periarterial basement membrane pathways. Acta Neuropathol..

[26] Yao Y., Chen Z.L., Norris E.H., Strickland S. (2014). Astrocytic laminin regulates pericyte differentiation and maintains blood brain barrier integrity. Nat. Commun..

[27] Pinotsi D., Rodighiero S., Campioni S., Csucs G. (2019). An Easy Path for Correlative Electron and Super-Resolution Light Microscopy. Sci. Rep..

[28] Haseleu J., Anlauf E., Blaess S., Endl E., Derouiche A. (2013). Studying subcellular detail in fixed astrocytes: Dissociation of morphologically intact glial cells (DIMIGs). Front. Cell. Neurosci..

[29] Cali C., Baghabra J., Boges D.J., Holst G.R., Kreshuk A., Hamprecht F.A., Srinivasan M., Lehvaslaiho H., Magistretti P.J. (2016). Three-dimensional immersive virtual reality for studying cellular compartments in 3D models from EM preparations of neural tissues. J. Comp. Neurol..

[30] Zhao Z., Nelson A.R., Betsholtz C., Zlokovic B.V. (2015). Establishment and Dysfunction of the Blood-Brain Barrier. Cell.

[31] Bell R.D., Winkler E.A., Singh I., Sagare A.P., Deane R., Wu Z., Holtzman D.M., Betsholtz C., Armulik A., Sallstrom J. (2012). Apolipoprotein E controls cerebrovascular integrity via cyclophilin A. Nature.

[32] Koizumi K., Hattori Y., Ahn S.J., Buendia I., Ciacciarelli A., Uekawa K., Wang G., Hiller A., Zhao L., Voss H.U. (2018). Apoepsilon4 disrupts neurovascular regulation and undermines white matter integrity and cognitive function. Nat. Commun..

[33] Poschl E., Schlotzer-Schrehardt U., Brachvogel B., Saito K., Ninomiya Y., Mayer U. (2004). Collagen IV is essential for basement membrane stability but dispensable for initiation of its assembly during early development. Development.

[34] Horsburgh K., Wardlaw J.M., van Agtmael T., Allan S.M., Ashford M.L.J., Bath P.M., Brown R., Berwick J., Cader M.Z., Carare R.O. (2018). Small vessels, dementia and chronic diseases - molecular mechanisms and pathophysiology. Clin. Sci..

[35] Shi Y., Yamada K., Liddelow S.A., Smith S.T., Zhao L., Luo W., Tsai R.M., Spina S., Grinberg L.T., Rojas J.C. (2017). ApoE4 markedly exacerbates tau-mediated neurodegeneration in a mouse model of tauopathy. Nature.

[36] Sbrana T., Ahluwalia A. (2012). Engineering Quasi-Vivo in vitro organ models. Adv. Exp. Med. Biol..

[37] Sun Y., Wu S., Bu G., Onifade M.K., Patel S.N., LaDu M.J., Fagan A.M., Holtzman D.M. (1998). Glial fibrillary acidic protein-apolipoprotein E (apoE) transgenic mice: Astrocyte-specific expression and differing biological effects of astrocyte-secreted apoE3 and apoE4 lipoproteins. J. Neurosci..

[38] Morikawa M., Fryer J.D., Sullivan P.M., Christopher E.A., Wahrle S.E., DeMattos R.B., O’Dell M.A., Fagan A.M., Lashuel H.A., Walz T. (2005). Production and characterization of astrocyte-derived human apolipoprotein E isoforms from immortalized astrocytes and their interactions with amyloid-beta. Neurobiol. Dis..

[39] Verghese P.B., Castellano J.M., Garai K., Wang Y., Jiang H., Shah A., Bu G., Frieden C., Holtzman D.M. (2013). ApoE influences amyloid-beta (Abeta) clearance despite minimal apoE/Abeta association in physiological conditions. Proc. Natl. Acad. Sci. USA.

[40] Collins-Praino L.E., Francis Y.I., Griffith E.Y., Wiegman A.F., Urbach J., Lawton A., Honig L.S., Cortes E., Vonsattel J.P., Canoll P.D. (2014). Soluble amyloid beta levels are elevated in the white matter of Alzheimer’s patients, independent of cortical plaque severity. Acta Neuropathol. Commun..

[41] Brinker T., Stopa E., Morrison J., Klinge P. (2014). A new look at cerebrospinal fluid circulation. Fluids Barriers Cns.

[42] Mazzei D., Guzzardi M.A., Giusti S., Ahluwalia A. (2010). A low shear stress modular bioreactor for connected cell culture under high flow rates. Biotechnol. Bioeng..

[43] Schneider C.A., Rasband W.S., Eliceiri K.W. (2012). NIH Image to ImageJ: 25 years of image analysis. Nat. Methods.

